# Plasma Inorganic Pyrophosphate Deficiency Links Multiparity to Cardiovascular Disease Risk

**DOI:** 10.3389/fcell.2020.573727

**Published:** 2020-12-09

**Authors:** Almudena Veiga-Lopez, Visalakshi Sethuraman, Nastassia Navasiolava, Barbara Makela, Isoken Olomu, Robert Long, Koen van de Wetering, Ludovic Martin, Tamas Aranyi, Flora Szeri

**Affiliations:** ^1^Department of Pathology, The University of Illinois at Chicago, Chicago, IL, United States; ^2^Department of Animal Science, Michigan State University, East Lansing, MI, United States; ^3^Department of Pediatrics and Human Development, Michigan State University, Lansing, MI, United States; ^4^Department of Pediatrics, Texas Tech University Health Sciences Center, Odessa, TX, United States; ^5^PXE Reference Centre (MAGEC Nord), University Hospital of Angers, Angers, France; ^6^Department of Obstetrics and Gynecology, Sparrow Hospital, Lansing, MI, United States; ^7^Department of Dermatology and Cutaneous Biology, PXE International Center of Excellence in Research and Clinical Care, Thomas Jefferson University, Philadelphia, PA, United States; ^8^Department of Molecular Biology, Semmelweis University, Budapest, Hungary; ^9^Institute of Enzymology, Research Centre for Natural Sciences, Budapest, Hungary; ^10^Department of Biochemistry, Semmelweis University, Budapest, Hungary

**Keywords:** cardiovascular disease risk, ectopic mineralization, alkaline phosphatase, pregnancy, pseudoxanthoma elasticum, vascular calcification, plasma inorganic pyrophosphate, sheep

## Abstract

Epidemiological studies indicate that elevated alkaline phosphatase activity is associated with increased cardiovascular disease risk. Other epidemiological data demonstrate that mothers giving multiple childbirths (multipara) are also at increased risk of developing late-onset cardiovascular disease. We hypothesized that these two associations stem from a common cause, the insufficient plasma level of the ectopic mineralization inhibitor inorganic pyrophosphate, which is a substrate of alkaline phosphatase. As alkaline phosphatase activity is elevated in pregnancy, we hypothesized that pyrophosphate concentrations decrease gestationally, potentially leading to increased maternal vascular calcification and cardiovascular disease risk in multipara. We investigated plasma pyrophosphate kinetics pre- and postpartum in sheep and at term in humans and demonstrated its shortage in pregnancy, mirroring alkaline phosphatase activity. Next, we tested whether multiparity is associated with increased vascular calcification in pseudoxanthoma elasticum patients, characterized by low intrinsic plasma pyrophosphate levels. We demonstrated that these patients had increased vascular calcification when they give birth multiple times. We propose that transient shortages of pyrophosphate during repeated pregnancies might contribute to vascular calcification and multiparity-associated cardiovascular disease risk threatening hundreds of millions of healthy women worldwide. Future trials are needed to assess if gestational pyrophosphate supplementation might be a suitable prophylactic treatment to mitigate maternal cardiovascular disease risk in multiparous women.

## Introduction

Recent epidemiological studies demonstrated that multiparous women have an increased risk for developing cardiovascular disease manifesting decade(s) after their pregnancies (Sanghavi et al., 2016; Kim et al., 2018; Oliver-Williams et al., 2019; Tanigawa et al., 2019). The molecular mechanism underlying this severe health-care problem is still elusive. Interestingly, in independent epidemiological studies high alkaline phosphatase levels were associated with coronary artery calcification (Panh et al., 2017), and future incidence of cardiovascular disease (Kunutsor et al., 2015; Kabootari et al., 2018) especially in young (<55 years of age) patients (Li et al., 2014; Rahmani et al., 2019). Alkaline phosphatase is the major degrading enzyme of inorganic pyrophosphate, a potent inhibitor of vascular calcification. Alkaline phosphatase activity is known to increase during pregnancy (Sussman et al., 1968; Okamoto et al., 1990). Therefore, we hypothesized that inorganic pyrophosphate concentration declines during pregnancy, due to increased alkaline phosphatase activity, contributing to the development of pregnancy-related late-onset maternal cardiovascular disease. According to our hypothesis, cardiovascular disease risk emerging in the two sets of independent epidemiological studies stems from a common cause, insufficient plasma inorganic pyrophosphate concentration.

Circulation provides precursors for the formation and remodeling of bones, a process under tight spatiotemporal regulation. Calcifying agents are present at high concentrations in the circulation, ready to start and propagate mineralization. Meanwhile, a network of active processes prevents the mineralization of arterial walls and other soft tissues (Back et al., 2018). One key anti-mineralization factor is inorganic pyrophosphate (Li et al., 2016a; Favre et al., 2017), present in the plasma at low micromolar concentrations and reduced in rare recessive ectopic mineralization disorders, such as generalized arterial calcification of infancy (GACI) and pseudoxanthoma elasticum (PXE). GACI is mostly caused by loss-of-function mutations of the gene coding for ectonucleotide pyrophosphatase phosphodiesterase 1 (ENPP1), which converts extracellular ATP into AMP and inorganic pyrophosphate. Lack of functional ENPP1 leads to the virtual absence of systemic inorganic pyrophosphate (Rutsch et al., 2003) resulting in severe *in utero* onset arterial calcification with high mortality. The majority of plasma inorganic pyrophosphate is generated from ATP provided by ABCC6 expressed primarily in hepatocytes (Jansen et al., 2013, 2014; Pomozi et al., 2013). Biallelic inactivating mutations of ABCC6 cause PXE, a disease characterized by reduced plasma inorganic pyrophosphate concentrations and milder vascular calcification compared to GACI.

We previously demonstrated the crucial role of inorganic pyrophosphate in preventing vascular calcification in the mouse models of PXE and GACI (Dedinszki et al., 2017). Interestingly, in the GACI mouse model, treating dams solely during gestation with inorganic pyrophosphate was sufficient to alleviate vascular calcification in their offspring (Dedinszki et al., 2017). Furthermore, ectopic calcification in PXE and GACI mouse models is effectively prevented by an inorganic pyrophosphate analog bisphosphonate (Li et al., 2016b, 2018). In both PXE and GACI, mothers are heterozygous carriers of the mutations without any apparent clinical phenotype. The current treatment for GACI in humans is postnatal bisphosphonates, increasing patient survival. In one case report, bisphosphonate treatment of a pregnant mother led to reduced vascular calcification in the fetus (Bellah et al., 1992). Altogether, the efficiency of maternal treatment during pregnancy to rescue vascular calcification in the offspring has been demonstrated in mice and humans. The efficacy of maternal *in utero* treatment with inorganic pyrophosphate and bisphosphonates in preventing vascular calcification of the F1 generation is indicative of insufficient maternal gestational inorganic pyrophosphate levels.

Our current work aims to demonstrate that inorganic pyrophosphate levels decline in healthy pregnancy, and low inorganic pyrophosphate concentrations may contribute to increased vascular calcification and thus to elevated cardiovascular disease risk. We propose that inorganic pyrophosphate supplementation during pregnancy could be a potential therapeutic approach to circumvent pregnancy-related late-onset maternal cardiovascular disease.

## Methods

### Animal Studies

The study was conducted at the MSU Sheep Teaching and Research Center. Eight sheep (Polypay × Dorset), pregnant with twins, were studied from gestational day (GD) 55 to postpartum day (PD) 40. Blood samples were collected between 8 and 10 am except for postnatal day 1, which was collected at 2.8 ± 3.8 h post-delivery. Eight non-pregnant adult sheep (Polypay × Dorset) were used as control. Pregnant sheep were housed indoors and their total mixed ration adjusted to energy requirements for pregnancy and lactation (National Research Council, 2007) as previously described (Gingrich et al., 2019). Offspring were housed with their mothers. After postnatal day 7, offspring had *ad libitum* access to grain creep feed. Non-pregnant sheep were kept outdoors on pasture with shade. All animals had *ad libitum* access to water. One female lamb in trio 4 was euthanized at postnatal day 15 due to an infection.

### Healthy Human Subjects

Blood samples were obtained from healthy pregnant women (20–40 years of age) undergoing scheduled Cesarean section at Sparrow Hospital, Lansing, MI, United States. Exclusion criteria included pregnancy complications, such as preterm birth, preeclampsia, and gestational diabetes. Blood samples from healthy non-pregnant women (20–40 years of age) were also collected. Exclusion criteria included calcification, bone or rheumatological disorder, cancer, diabetes or hypertension.

### Pseudoxanthoma Elasticum Patients

The study was part of the phenotyping in the French PXE cohort (ClinicalTrials.gov, NCT01446380) from 2008 to October 25, 2019. PXE diagnosis was confirmed on distinctive skin lesions, dermal elastorrhexis, ophthalmological signs and/or two mutations of the ABCC6 gene (Legrand et al., 2017). Lower limb artery calcification was determined using unenhanced computed tomography (CT) as described by Chowdhury et al. (2017). Arterial tree of each lower limb was divided into aortoiliac, femoro-popliteal, and crural segments and scored. Lower limb artery calcification was calculated as the sum of all segmental scores of both legs normalized by the total arterial lengths.

### Platelet Free Plasma Preparation and Inorganic Pyrophosphate Determination

Blood was collected in Vacutainer CTAD tubes with 50 μl 15% K_3_EDTA, as described previously (Dedinszki et al., 2017). Plasma was separated (1,000 × *g* 4°C, 10-min centrifugation), and platelet-free plasma was prepared (2,200 × *g* 4°C 30-min centrifugation in Centristart^®^ 300.000 MW filter tubes). First, inorganic pyrophosphate was converted into ATP by ATP sulfurylase (NEB) in the presence of APS, and then ATP was determined using bioluminescence (BacTiter Glo, Promega). inorganic pyrophosphate concentration was calculated using standards and correction for ATP concentrations (Dedinszki et al., 2017). Samples with hemolysis were excluded.

### Statistics

Data were analyzed using GraphPad Prism 8.3.0. and, depending on normality, shown with means ± SD or medians and 25th–75th interquartiles. We used unpaired two-tailed *t*-test with Welch’s correction for healthy women and unpaired non-parametric two-tailed Mann-Whitney test for PXE patients except for age in PXE patients where significance was determined using a two-tailed unpaired *t*-test. Animal groups were compared using multiple comparisons of the paired data with mixed effect models not assuming sphericity and concomitant Sidak’s multiple comparisons. Significance was accepted at *p* < 0.05.

### Study Approval

All sheep procedures were approved by the Institutional Animal Care and Use Committee of Michigan State University. Euthanasia was conducted on one animal due to an injury-related infection independent of the present study. The euthanasia was performed by a Campus Animal Resource (CAR) veterinarian via a barbiturate overdose as per the American Medical Veterinarian Association (AMVA) guidelines. All procedures on healthy human individuals at Sparrow Hospital and Michigan State University (IRB: 17-1359) was approved by the Institutional Review Board. All procedures on PXE patients were approved by the Institutional Research Committee of the University Hospital of Angers. The study on PXE patients was performed as a part of the protocol of phenotyping of French PXE cohort (ClinicalTrials.gov, Identifier: NCT01446380). All procedures involving human participants were in accordance with the ethical principles of the Helsinki declaration. Written informed consent was obtained from all participants prior to inclusion in the study.

## Results

### Plasma Inorganic Pyrophosphate Concentration Changes Dynamically During Pre- and Postpartum in Sheep

**FIGURE 1:**
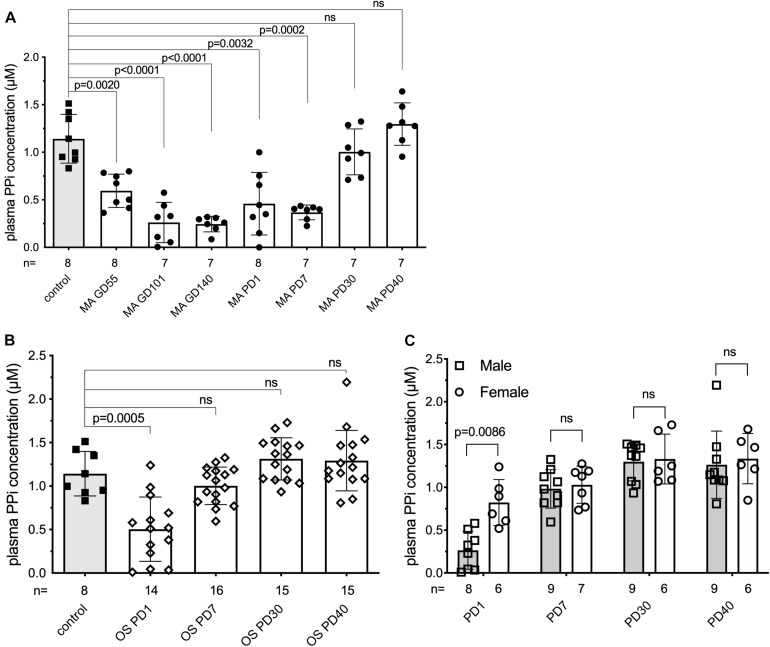
Plasma inorganic pyrophosphate changes dynamically in sheep pre- and postpartum. **(A)** Plasma inorganic pyrophosphate concentration of pregnant sheep between gestational day 55 and postpartum day 40 (*n* = 8, closed circles, white bars) compared to non-pregnant adult females (*n* = 8, closed squares, gray bar). **(B)** Plasma inorganic pyrophosphate concentration of lambs born from sheep in panel **(A)** between postnatal day 1–40 (weaning) (*n* = 16, open diamonds, white bars) compared to the non-pregnant adult females (*n* = 8, closed squares, gray bar, from panel **A**). **(C)** Plasma inorganic pyrophosphate concentration of male (*n* = 9, open squares, gray bars) and female (*n* = 7, open circles, white bars) lambs from panel **(B)**. MA, mother; OS, offspring; GD, gestational day; PD, postpartum/postnatal day. Values for the data depicted were determined in experiments using at least three technical replicates. Data were analyzed using GraphPad Prism 8.3.0. Animal groups were compared using multiple comparisons of the paired data with mixed effect models not assuming sphericity. Concomitant Sidak’s multiple comparison tests were performed to assess significance. A female lamb was euthanized at postnatal day 15, due to a reason unrelated to the study. Samples that showed hemolysis were not processed further and excluded from the evaluation. The exact number of samples determined for each timepoint are presented under the corresponding bars. Data values are represented as means ± SD. Significance was accepted at *p* < 0.05. PPi, inorganic pyrophosphate.

As alkaline phosphatase activity is known to increase during pregnancy and inorganic pyrophosphate is an established substrate of alkaline phosphatase, we hypothesized that plasma inorganic pyrophosphate concentrations are decreased during pregnancy. Therefore, we sought to study plasma inorganic pyrophosphate kinetics pre- and postpartum in an animal model. Since current inorganic pyrophosphate determinations require high blood volumes relative to rodent size, sheep were chosen for their larger body weight and longer gestation (∼147 days). This allowed multiple samplings in a longitudinal study to follow plasma inorganic pyrophosphate concentrations. Non-pregnant control sheep had a plasma inorganic pyrophosphate level of 1.14 ± 0.26 μM (Figure 1A), similar to that of rodents and humans (Dedinszki et al., 2017). This control group was compared to a cohort of sheep pregnant with twins, monitored throughout gestation and lactation. We observed a progressive decrease of plasma inorganic pyrophosphate concentrations during pregnancy, with a nadir at gestational day 140, shortly before term. Variable maternal inorganic pyrophosphate concentrations were detected on the day of birth and levels gradually increased postpartum, reaching the plasma inorganic pyrophosphate levels of control sheep by postpartum day 30.

We also monitored the plasma inorganic pyrophosphate concentration in the 16 newborn lambs on the same postnatal days (Figure 1B). Similar to their mothers, the newborns had variable but low plasma inorganic pyrophosphate concentrations on postnatal day 1. Within 7 days after birth, plasma inorganic pyrophosphate levels increased to concentrations detected in non-pregnant adult sheep. On postnatal day 1, males had lower inorganic pyrophosphate concentrations than females (0.26 ± 0.22 and 0.82 ± 0.27 μM, respectively), but this sex-dependent difference disappeared by postnatal day 7 (Figure 1C). Within sheep families (trios constituted by a mother and its twins), inorganic pyrophosphate kinetics showed a similar pattern, with dams lagging behind their lambs (Supplementary Figure 1).

### Plasma Inorganic Pyrophosphate Concentration Is Decreased in Healthy Human Pregnancy

**FIGURE 2:**
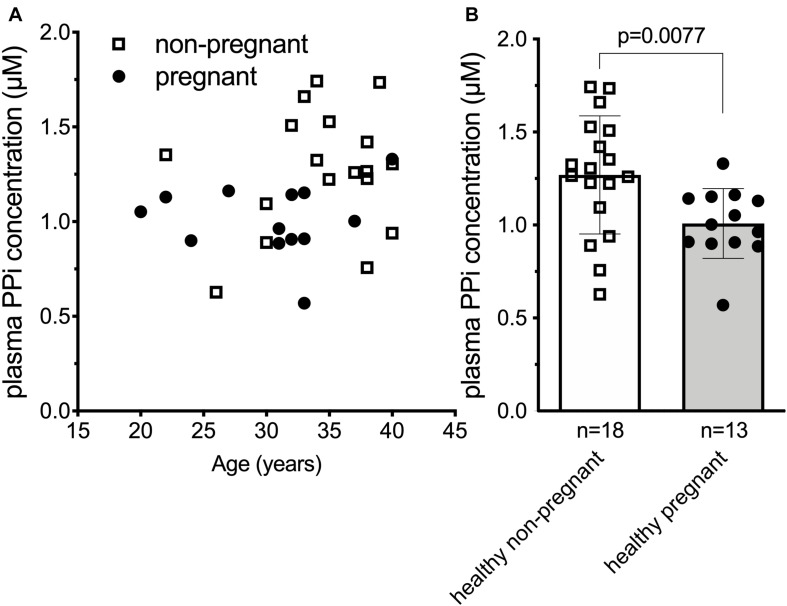
Plasma inorganic pyrophosphate concentration is significantly decreased in uncomplicated human pregnancy. **(A)** Plasma inorganic pyrophosphate concentration versus age in pregnant (*n* = 13, closed circles) and non-pregnant (*n* = 18, open squares) healthy women. **(B)** Plasma inorganic pyrophosphate concentration of healthy pregnant women (*n* = 13, closed circles, gray bar) at term versus non-pregnant healthy women (*n* = 18, open squares, white bar). Values for the data depicted were determined at least in two independent experiments using at least three technical replicates. Data were analyzed using GraphPad Prism 8.3.0. Data in panel **(B)** followed normal distribution according to Kolmogorov-Smirnov test. We used unpaired two-tailed *t* test with Welch’s correction not assuming equal SDs to determine significance. Bars represent means ± SD. Significance was accepted at *p* < 0.05. PPi, inorganic pyrophosphate.

Our results in the sheep suggested that the plasma inorganic pyrophosphate decrease during pregnancy might be a general physiological phenomenon relevant to other species as well. To test this hypothesis in humans, we compared plasma inorganic pyrophosphate concentrations between non-pregnant (*n* = 18; age = 34.6 ± 4.9 year) and pregnant women (*n* = 13; age = 30.4 ± 5.6 year) at term before their scheduled Cesarean-section. Healthy pregnant women had significantly lower plasma inorganic pyrophosphate concentrations than non-pregnant women (1.01 ± 0.19 and 1.28 ± 0.33 μM, respectively) (Figure 2).

### Cardiovascular Disease Risk Associated Vascular Calcification Is Increased in Pseudoxanthoma Elasticum Patients With Multiple Childbirths

Epidemiological data of thousands of healthy individuals indicate that multiparous women have an elevated risk for cardiovascular disease (Ness et al., 1993; Sanghavi et al., 2016; Kim et al., 2018; Oliver-Williams et al., 2019; Tanigawa et al., 2019) and stroke (Qureshi et al., 1997). Given our findings, we hypothesized that multiple pregnancies increase cardiovascular disease risk due to repeated, yet transitory decrease in the concentration of the mineralization inhibitor, inorganic pyrophosphate.

**FIGURE 3:**
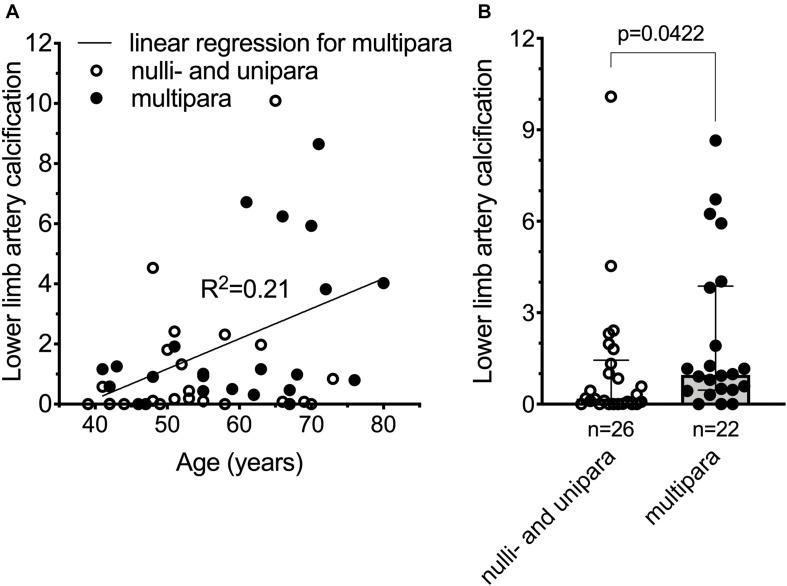
Cardiovascular disease risk is increased in pseudoxanthoma elasticum patients giving multiple (>2) childbirths. **(A)** Normalized lower limb artery calcification (nLLAC) score versus age in nulli/unipara (*n* = 26, open circles) and in multipara (*n* = 22, closed circles) with the result of a simple linear regression performed for nLLAC score versus age in multipara. **(B)** nLLAC score is higher in multiparous PXE patients (*n* = 22, closed circles, gray bar) compared to nulli/uniparous PXE patients (*n* = 26, open circles, white bar). The 48 year-old nulliparous subject with renal amyloidosis had an LLAC and nLLAC of 6,955.4 and 4.54, respectively. Data were analyzed using GraphPad Prism 8.3.0. According to Kolmogorov-Smirnov test data for panel **(B)** did not follow normal distribution. We used unpaired non-parametric two-tailed Mann-Whitney test to analyze significance. Bars represent medians with 25th–75th interquartiles. Significance was accepted at *p* < 0.05.

Pseudoxanthoma elasticum patients have deleterious mutations that lead to non-functional ABCC6 resulting in chronically low plasma inorganic pyrophosphate concentrations and, as a consequence, increased vascular calcification. PXE patients also have higher risk of developing cardiovascular disease and stroke (Trip et al., 2002; Köblös et al., 2010; Kauw et al., 2017; De Vilder et al., 2018), although the latter is debated (Hornstrup et al., 2011). We postulated, therefore, that PXE patients with *ab ovo* low inorganic pyrophosphate levels, giving birth multiple times, might have even more pronounced vascular calcification, further increasing their risk to develop cardiovascular disease. We tested our hypothesis in a cohort of PXE patients (187 individuals, 122 females), where clinical data, including the cardiovascular disease risk factor lower limb artery calcification (Chowdhury et al., 2017) was studied. To assess the association between number of childbirths to cardiovascular disease risk, we selected women over 40 years of age with available information on parity and lower limb artery calcification score. There was no reported hepatic pathology, gestational diabetes, or chronic kidney disease in any of the study subjects. One nulliparous patient was presented with renal amyloidosis but without any other cardiovascular risk factor. To better distinguish patients with no or low numbers of childbirths from those of higher number of childbirths, we excluded patients with two childbirths, and dichotomized the remaining patients to nulli/uniparous (*n* = 26; age = 54.8 ± 9.7 year) and multiparous (>2 childbirths, *n* = 22; age = 59.5 ± 11.6 year) subgroups (age, *p* = 0.1279). The BMI (kg/m^2^) values (24.9 ± 5.3 and 24.7 ± 4.2), the incidence of high blood cholesterol (30.8% ± 1.8 and 27.3% ± 2.1) and smoking (23.1% ± 1.7 and 22.8% ± 1.9) did not differ in the nulli/unipara and multipara groups, respectively. Amongst our included patients (Supplementary Table 1), the lower limb artery calcification score normalized for arterial length did not correlate with age in nulli/unipara, and weakly correlated with age in multipara (Figure 3A). Moreover, multiparous PXE patients had a significantly higher normalized lower limb artery calcification score compared to nulli/uniparous patients (*p* < 0.05) (Figure 3B). Thus, in these PXE patients, parity positively correlated with elevated vascular calcification, which is known to be linked to an increase in cardiovascular disease risk.

## Discussion

### Inorganic Pyrophosphate Dynamics Correlate to Alkaline Phosphatase Kinetics Reported in the Literature

Inorganic pyrophosphate is an important substrate of tissue non-specific alkaline phosphatase (TNAP) (Whyte et al., 1995), which cleaves inorganic pyrophosphate into inorganic phosphate (Pi). According to the sheep gene expression atlas dataset^1^, TNAP is highly expressed in the placenta. Moreover, TNAP expression and alkaline phosphatase activity are dramatically elevated in sheep during pregnancy (Healy, 1971; Clark et al., 2017; Zywicki et al., 2018). The gradual gestational inorganic pyrophosphate decline we observed in sheep aligns well with these reports. Similarly, the increase in the maternal plasma inorganic pyrophosphate concentration we observed postpartum corresponds well with the decline of alkaline phosphatase activity after parturition (Bekeova et al., 1993). Additionally, the increase in inorganic pyrophosphate concentration in newborns aligns with the drop in serum alkaline phosphatase activity shown in lambs (Healy, 1971). Collectively, the alkaline phosphatase kinetics reported in the literature provides a plausible explanation for the dynamic plasma inorganic pyrophosphate changes we observed in sheep.

In humans TNAP plays a pivotal role in bone metabolism by degrading the mineralization inhibitor inorganic pyrophosphate. TNAP defects cause hypophosphatasia, a severe skeletal/neural disorder. Three carriers of mutant *ALPL* alleles coding for TNAP had *per se* decreased TNAP and consequently increased plasma inorganic pyrophosphate levels, the latter normalized during pregnancy (Whyte et al., 1995). In parallel, the expression of placental alkaline phosphatase (PALP), encoded by a hominid-specific different gene, increased. As PALP also degrades inorganic pyrophosphate (Whyte et al., 1995), the normalization of inorganic pyrophosphate levels in the carriers was likely due to emerging PALP activity. In healthy pregnancies, PALP expression gradually rises and peaks at term (Sussman et al., 1968; Okamoto et al., 1990). As a result, total serum alkaline phosphatase activity increases approximately two-fold (Hirano et al., 1987). Taken together, gestationally elevated PALP activity serves as a plausible explanation for the decreased plasma inorganic pyrophosphate concentrations we observed in healthy pregnant women.

The evolutionary advantage of increased gestational alkaline phosphatase activity is elusive, but it likely promotes fetal development and growth (She et al., 2000; Liu et al., 2018). The resulting decreased concentration of inorganic pyrophosphate is compensated for in the healthy fetus, while elevated maternal cardiovascular disease risk emerges only in the long term and most likely does not affect evolutionary fitness.

### Plasma Inorganic Pyrophosphate Deficiency Is the Potential Link Between Alkaline Phosphatase Levels and Multiple Pregnancies, Both Contributing to Cardiovascular Disease Risk

High alkaline phosphatase levels were previously associated with coronary artery calcification in 500 patients (Panh et al., 2017). Moreover, in two prospective studies involving large cohorts of healthy individuals, baseline alkaline phosphatase levels correlated to the future incidence of cardiovascular disease independent of traditional cardiovascular disease risk factors (Kunutsor et al., 2015; Kabootari et al., 2018). Finally, in meta-analyses of ∼20,000 participants from 7 studies and ∼150,000 participants from 24 studies, elevated alkaline phosphatase activity was associated with increased cardiovascular disease mortality, especially in young patients (<55 years of age) (Li et al., 2014; Rahmani et al., 2019). On the other hand, several epidemiological studies established that multiparous women also have increased risk for developing cardiovascular disease decade(s) after their pregnancies (Sanghavi et al., 2016; Kim et al., 2018; Oliver-Williams et al., 2019; Tanigawa et al., 2019).

Our data suggest that recurrent deficiency in maternal plasma inorganic pyrophosphate concentration, likely caused by elevated PALP activity over subsequent pregnancies, is a plausible mechanism for the increased cardiovascular disease risk in multiparous women. In the above epidemiological studies high number of subjects were studied to show the association of cardiovascular disease risk to the number of childbirths and, in an independent set of studies, the cardiovascular disease risk to intrinsically high alkaline phosphatase activity. However, we were able to show the correlation of multiple childbirths to increased lower limb vascular calcification, an established cardiovascular disease risk factor, in our limited set of patients with a rare disease characterized by pathological low plasma inorganic pyrophosphate levels. This suggests that plasma inorganic pyrophosphate concentration is likely to play a pivotal role in the etiology of the parity-dependent cardiovascular disease-risk. As the mineralization inhibitor inorganic pyrophosphate is the substrate of alkaline phosphatase and plasma inorganic pyrophosphate concentrations decline during pregnancy meanwhile alkaline phosphatase increases, it therefore seems justified to conclude, that the low gestational inorganic pyrophosphate level is the consequence of the higher alkaline phosphatase activity, however to establish cause-effect relationship further direct evidence is required. Moreover, it is possible that compensatory mechanisms exist to counteract for the gestational drop in plasma pyrophosphate concentration, a question that further studies need to explore. Our results demonstrate for the first time, that the maternal plasma concentration of the major mineralization inhibitor inorganic pyrophosphate declines during healthy pregnancy, likely due to increased alkaline phosphatase activity. We hypothesize that the low level of inorganic pyrophosphate is the common denominator and putative causal link between the established epidemiological correlations of increased cardiovascular disease risk to multiple childbirths, on the one hand, and to elevated alkaline phosphatase activity, on the other. Finally, in PXE, a disorder with pathologically low plasma inorganic pyrophosphate levels, we demonstrated that multiple childbirths associated with increased lower limb vascular calcification, an established risk factor for cardiovascular disease. Based on our results and assumptions, restoration of sufficient inorganic pyrophosphate levels during pregnancy might be a potential prophylaxis/treatment to circumvent pregnancy-related maternal cardiovascular disease risk both in healthy women and in patients with compromised inorganic pyrophosphate levels. This treatment might also potentially benefit the children by reducing the prevalence of vascular calcification in the next generation. However, efficacy and safety of such application needs to be thoroughly evaluated in future translational studies.

## Data Availability Statement

The original contributions presented in the study are included in the article/Supplementary Material, further inquiries can be directed to the corresponding author/s.

## Ethics Statement

The studies involving human participants were reviewed and approved by Sparrow Hospital and Michigan State University Institutional Review Board (IRB: 17-1359) (healthy human individuals) and by the Institutional Research Committee of the Angers University Hospital (PXE patients). The study on PXE patients was performed as a part of the protocol of phenotyping of French PXE cohort (ClinicalTrials.gov, Identifier: NCT01446380). The patients/participants provided their written informed consent to participate in this study. The animal study was reviewed and approved by Institutional Animal Care and Use Committee of Michigan State University.

## Author Contributions

AV-L led/conceptualized and participated in animal and healthy human studies and contributed to the manuscript writing. VS, IO, and RL recruited the human healthy pregnant subjects and samples. NN determined the lower limb artery calcification. BM participated in sheep and healthy human studies. KW provided the reagents and equipment and contributed to the manuscript writing. LM provided the PXE patient data and discussed the results. TA conceptualized/supervised the project and contributed to the manuscript writing; FS conceptualized/supervised the project, performed the inorganic pyrophosphate experiments, analyzed the data and wrote the manuscript. All authors reviewed/edited the manuscript, approved the final version and agreed to be accountable for all aspects of the work in ensuring that questions related to the accuracy or integrity of any part of the work are appropriately investigated and resolved.

## Conflict of Interest

FS and KW are coinventors of the patent WO2018052290 “Oral pyrophosphate for use in reducing tissue calcification.” The remaining authors declare that the research was conducted in the absence of any commercial or financial relationships that could be construed as a potential conflict of interest. The reviewer OL declared a past collaboration with one of the authors LM to the handling editor.

## Footnotes

1 http://biogps.org/sheepatlas/
