# Supplementary material for: Plasma Inorganic Pyrophosphate Deficiency Links Multiparity to Cardiovascular Disease Risk
Source: Front Cell Dev Biol. 2020 Dec 9;8:573727. doi: 10.3389/fcell.2020.573727 (PMC7755719; doi:10.3389/fcell.2020.573727)
Supplement: Supplementary file 1 [file Data_Sheet_1.PDF]

frontiers

frontiers

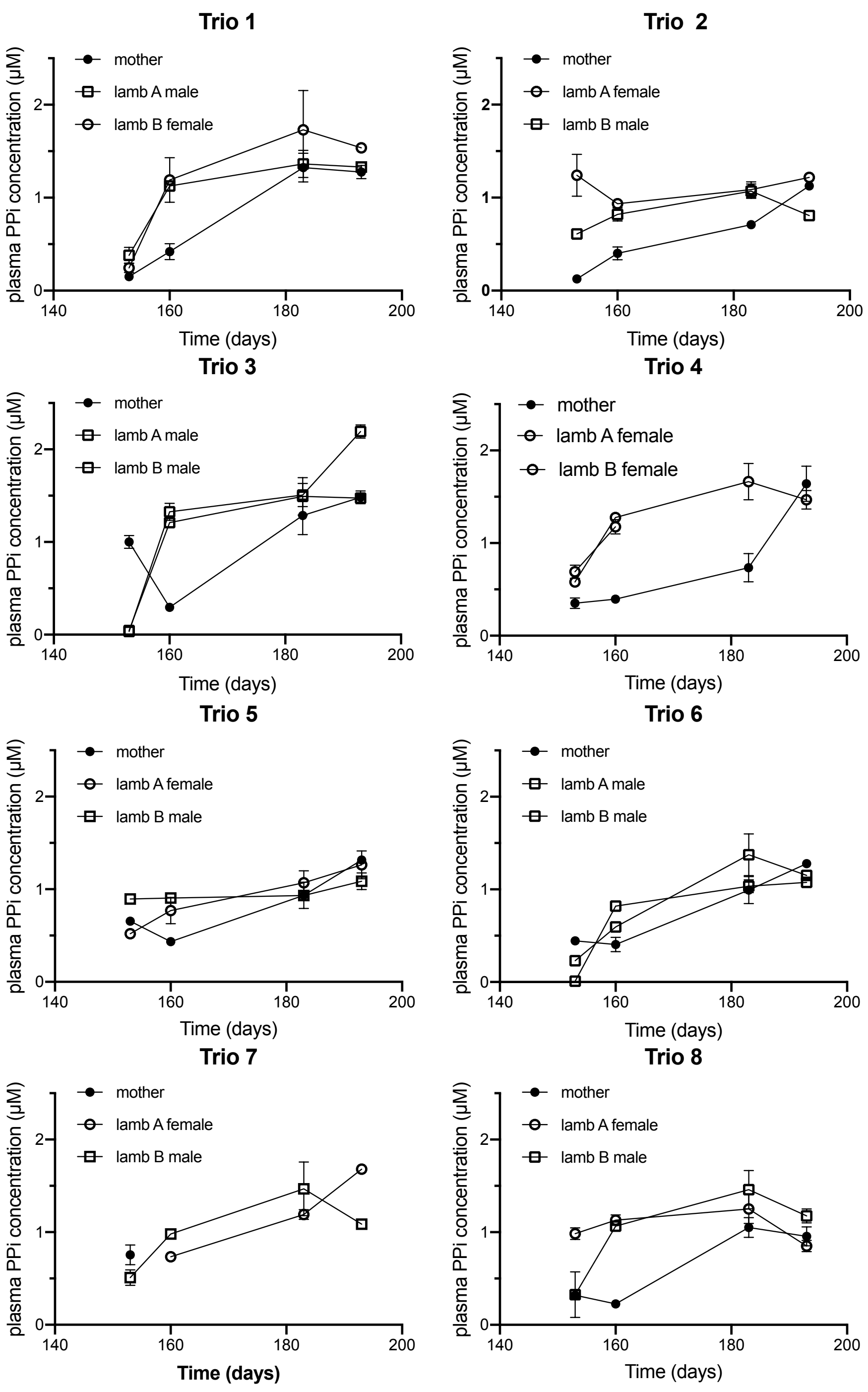

Supplementary Figure
Plasma inorganic pyrophosphate concentration of sheep trios compiled of mothers and their respective lambs (from Figure1) from postnatal day 1 to weaning. A female lamb from trio 4. was euthanized at postnatal day 15, unrelated to the study. Samples that showed hemolysis were not processed further. Values for the data depicted were determined in experiments using at least 3 technical replicates. Data values are represented as mean ± SD.

ID	Age (y)	childbirth number	LLAC	nLLAC	Allele 1	Allele 2	Phenodex score
1	58	0	0	0	n.a.	n.a.	S1E3G0V0C0R0
2	48	0	6955.4	4.54	p.Gly1042Ser	p.Gly1042Ser	S3E2G0V1C1R0
3	49	0	4.9	0.003	del 23-29	n.a.	S3E3G0V1C0R0
4	51	0	3476.2	2.42	p.Arg518Gln	p.Arg1138Gln	S2E2G0V2C0R0
5	42	0	14.6	0.01	del23-29	p.Arg1141Ter	S2E2G0V1C0R1
6	41	0	813.1	0.58	n.a.	n.a.	S3E3G0V2C0R1
7	48	0	180.9	0.11	n.a.	n.a.	S2E2G0V1C0R0
8	39	0	0	0	p.Thr364Arg	IVS26-1 G>A	S2E2G0V1C1R0
9	44	0	0	0	p.Arg1114His	p.Arg1114His	S1E2G1V0C0R1
10	70	0	0	0	p.Arg1164Gln	n.a.	S3E2G0V1C1R0
11	52	0	2132.2	1.33	p.Arg518Ter	not identified	S3E3G0V1C0R1
12	63	1	3221	1.98	p.Arg1141Ter	p.Arg1141Ter	S3E3G0V2C0R1
13	53	1	296	0.19	n.a.	n.a.	S3E3G0V1C0R0
14	55	1	1495.2	1.01	n.a.	n.a.	S3E3G0V1C0R0
15	55	1	163	0.1	p.Arg1141Ter	p.Arg1141Ter	S3E3G0V0C0R1
16	69	1	117	0.08	n.a.	n.a.	S1E3G0V0C0R1
17	65	1	14166.4	10.09	p.Arg1138Trp	p.Arg1141Ter	S3E3G0V2C1R1
18	62	1	490.5	0.32	n.a.	n.a.	S3E3G0V1C0R1
19	51	1	267.9	0.17	n.a.	n.a.	S3E2G0V1C0R1
20	53	1	740.1	0.45	p.Arg1221Cys	del30-31	S3E3G0V1C2R1
21	66	1	123.6	0.08	n.a.	n.a.	S3E2G0V0C0R1
22	73	1	1338.2	0.84	p.Arg1141Ter	IVS8+2 delTG	S3E3G0V3C1R0
23	67	1	0	0	del23-29	p.Arg391Gly	S3E2G0V2C0R0
24	42	1	0	0	p.Arg1141Ter	600+1G>A	S3E2G0V1C0R1
25	50	1	2547.3	1.81	delABCC6	n.a.	S3E3G0V2C1R1
26	58	1	3563.1	2.32	Val787Ile; Gly755Arg	p.Arg1164Ter	S3E3G1V3C0R1
27	67	3	719.1	0.47	p.Arg487Gln	998+2_998+3delTG	S0E3G0V1C0R0
28	55	3	1427.9	0.94	c.36+1G>C	p.Gln378Ter	S3E2G0V1C0R0
29	42	3	911.4	0.59	p.Arg518Gln	not identified	S1E2G0V3C0R1
30	55	3	656.5	0.44	n.a.	n.a.	S3E2G1V2C0R0
31	41	3	1830.4	1.16	p.Arg1114Cys	IVS26A(-2)->G(exon27)	S3E2G1V2C1R0
32	46	3	0	0	p.Tyr768Ter	p.Thr364Arg	S2E2G0V0C0R1
33	80	3	6225.5	4.02	n.a.	n.a.	S3E3G0V3C1R1
34	59	3	818	0.5	c.36+1G>C	c.1868-5T>G	S2E3G0V1C0R0
35	51	3	2925	1.92	IVS22-26_IVS22-4del	p.Trp140Leu	S2E2G0V2C2R0
36	61	3	10494	6.72	del 23_29	p.Gln1364Pro	S2E3G0V2C1R0
37	71	3	12487.5	8.65	c.2787+1G>T	Del exon 7	S3E3G0V3C1R0
38	70	3	9077.2	5.93	n.a.	n.a.	S1E3G0V3C0R0
39	68	3	1483.5	0.99	p.Thr1130Met	del23-29	S2E3G0V1C1R0
40	72	3	5702.2	3.83	p.Arg1138Trp	p.Arg1141Ter	S2E3G1V1C2R1
41	48	3	1444.2	0.91	p.Arg1141Ter	IVS21+1 G>T	S3E2G0V2C2R0
42	66	3	9646.7	6.24	p.Arg807Gln	p.Arg807Gln	S3E3G1V2C1R0
43	47	3	0	0	Leu780fs30(2338delC)	p.Arg1314Gln	S2E3G0V0C1R1
44	67	4	0	0	p.Gln715Argfs*12	n.a.	S0E3G0V0C1R0
45	62	4	468.1	0.31	p.Arg1030Ter	del23-28	S3E3G0V2C1R0
46	63	4	1803	1.17	1868-5T>G	p.Gln378Ter	S2E2G0V1C0R0
47	76	4	1190.6	0.8	p.Gly998Arg	n.a.	S2E3G0V1C1R0
48	43	4	1893.9	1.26	p.Glu1400Lys	n.a.	S2E2G0V1C0R1

Supplementary Table

Pseudoxanthoma elasticum patient characteristics, genotype and phenotype. Lower limb artery calcification (LLAC) was determined using unenhanced computed tomography (Chowdhury et al. 2017). Normalized lower limb artery calcification (nLLAC) is the total lower limb calcification score normalized by total arterial lengths. Genotype was determined by exome sequencing. Modified

Phenodex index (Legrand et al. 2017) is provided for six organ systems: skin (S), eye (E) gastrointestinal (G), vascular (V), cardiac (C) and renal (R).

References:

Chowdhury, M. M., G. C. Makris, J. M. Tarkin, F. R. Joshi, P. D. Hayes, J. H. F. Rudd, and P. A. Coughlin. 2017. "Lower limb arterial calcification (LLAC) scores in patients with symptomatic peripheral arterial disease are associated with increased cardiac mortality and morbidity." *PLoS One* 12 (9):e0182952. doi: 10.1371/journal.pone.0182952.

Legrand, A., L. Cornez, W. Samkari, J. M. Mazzella, A. Venisse, V. Boccio, K. Auribault, B. Keren, K. Benistan, D. P. Germain, M. Frank, X. Jeunemaitre, and J. Albuisson. 2017. "Mutation spectrum in the ABCC6 gene and genotype-phenotype correlations in a French cohort with pseudoxanthoma elasticum." *Genet Med* 19 (8):909-917. doi: 10.1038/gim.2016.213.
